# The overexpression of glypican-5 promotes cancer cell migration and is associated with shorter overall survival in non-small cell lung cancer

**DOI:** 10.3892/ol.2013.1622

**Published:** 2013-10-11

**Authors:** YAN LI, LIYUN MIAO, HOURONG CAI, JINGJING DING, YONGLONG XIAO, JUN YANG, DEPING ZHANG

**Affiliations:** 1 Department of Respiratory Medicine, The Affiliated Drum Tower Hospital of Nanjing University Medical School, Nanjing, Jiangsu 210008, P.R. China; 2 Department of Pathology, The Affiliated Drum Tower Hospital of Nanjing University Medical School, Nanjing, Jiangsu 210008, P.R. China

**Keywords:** glypican-5, non-small cell lung carcinoma, survival, prognosis, metastasis

## Abstract

Although the correlation between glypican-5 (GPC5) and lung cancer is well known, the effect of GPC5 expression on non-small cell lung cancer (NSCLC) survival remains to be determined. In the present study, GPC5 expression in A549, H3255, and SPC-A1 NSCLC cell lines was evaluated by reverse transcription-polymerase chain reaction (RT-PCR) and western blot analysis. GPC5 mRNA and protein expression levels were found to be higher in A549 and H3255 cells compared with SPC-A1 cells. The role of GPC5 in NSCLC cell migration was evaluated *in vitro* by shRNA-mediated knockdown or the overexpression of GPC5 through scratch and transwell assays. The mean migration rates of cancer cells transfected with pRNAT-shRNA-GPC5-1 were reduced compared with the controls in A549 (P<0.001) and H3255 (P=0.001), while the migration rate of SPC-A1 with GPC5 overexpression was higher than that of the control (P=0.001). The downregulation of GPC5 impeded the transmigration of A549 and H3255 while the upregulation of GPC5 expression promoted the transmembrane invasion of SPC-A1. Furthermore, a panel of formalin-fixed paraffin-embedded NSCLC tissues from 127 patients undergoing curative resection (stages I, II and III) between January, 2003 and December, 2008 were obtained in order to investigate the correlation between GPC5 expression and clinicopathological factors using immunohistochemical methods. The results demonstrated that high GPC5 expression levels in NSCLC were associated with respiratory symptoms in lung cancer diagnosis, poor differentiation, vascular invasion, regional lymph node metastasis and a higher TNM stage. Using the Kaplan-Meier method, NSCLC patients with high levels of GPC5 expression demonstrated a significantly shorter overall survival time compared with those with low GPC5 expression levels (median postsurgical survival time: 14.0 months vs. 59.0 months, P=0.001). GPC5 expression was also identified as an independent prognostic factor by Cox regression analysis [adjusted hazard ratio: 2.18; 95% confidence interval (CI): 1.35–3.52; P=0.001]. This study suggested that increased levels of GPC5 expression are a poor prognostic marker for NSCLC.

## Introduction

Lung cancer is one of the leading causes of cancer mortality worldwide (1). Non-small cell lung cancer (NSCLC) accounts for ~85% of all lung cancer cases, with a five-year survival rate of 15.1% in China (2). The main cause of mortality for NSCLC is cancer metastasis, which is difficult to treat and is currently a popular area for lung cancer research. Despite maximal therapy, surgically treated patients with stage I–III NSCLC are at risk for developing metastatic disease. Clinicians and researchers should urgently explore treatment targets in order to inhibit cancer metastasis, thereby establishing more reliable and effective therapies for NSCLC.

Glypican-5 (GPC5) is one of the six members of the glypican family that are bound to the external surface of the plasma membrane by glycosyl-phosphatidylinositol (GPI) linkage (3). The GPC5 gene has eight exons encoding 572 amino acids and spans a large genomic region of 1.47 Mb at chromosome 13q31.3. GPC5 expression is developmentally regulated, with a general role in the control of growth and differentiation during mammalian development (4). A genome-wide association study (GWAS) has reported that genetic variations of GPC5 may contribute to an increased risk of lung cancer in patients who have never smoked (5). GPC5 has also been found to be abnormally expressed in various human tumors. Studies have shown that GPC5 expression was lower in tumors with a relatively better prognosis, while it was higher in tumors with greater metastatic potential, such as in small cell lung cancer and rhabdomyosarcoma (6–9). However, the effect of GPC5 expression on NSCLC prognosis has yet to be defined. We hypothesized that GPC5 affects the prognosis of NSCLC patients by involving the metastatic process.

The aim of the present study was to investigate the expression pattern of GPC5 in NSCLC cell lines and tumor tissue samples. The effects of GPC5 expression on cancer cell migration were assessed. The correlation of GPC5 expression levels with NSCLC survival was also examined.

## Materials and methods

### Cell culture, vector construction and plasmid transfection

The NSCLC cell lines A549, H3255 and SPC-A1 were cultured in RPMI-1640 (Wisent Inc., St- Bruno, QC, Canada) supplemented with 10% fetal bovine serum (HyClone, Logan, UT, USA), 100 U/ml penicillin and 100 μg/ml streptomycin at 37°C in an atmosphere of 5% CO_2_. Coding sequences of human GPC5 was cloned into pEGFP-N1 and designated as pEGFP/GPC5. GPC5 targeted three shRNAs, these were predicted to be 5′-GATCCGTTCGGAAACTTTTCCAGTTCAAGAGACTGGAAAAGTTTCCGAACTTTTTTTTCAA-3′, 5′-GATCCTTTGTAAACAGATTTTTTGTCAAGAGCAAAAAATCTGTTTACAAATTTTTTTTCAA-3′ and 5′-GATCCAAAGTTATACTCAGCGTGTTCAAGAGACACGCTGAGTATAACTTTTTTTTTTTCAA-3′. All three shRNAs targeting GPC5 were constructed into pRNAT-U6.1/Neo, as pRNAT-shRNA-GPC5-1,2,3. The cell lines A549, H3255 and SPC-A1 were transfected with pEGFP/GPC5, pEGFP-N1, pRNAT-shRNA-GPC5-1,2,3 or pRNAT-U6.1 using TurboFect (Fermentas, Vilnius, Lithuania) according to the manufacturer’s instructions. GPC5 expression was examined using reverse transcription-polymerase chain reaction (RT-PCR) and western blot analysis.

### RNA isolation, reverse transcription and PCR

Total RNA was extracted using TRIzol reagent (Invitrogen, Carlsbad, CA, USA) and reverse transcribed into cDNA using a PrimeScript RT reagent kit (Takara, Dalian, China) according to the manufacturer’s instructions. PCR was performed using the Mastercycler Gradient (Eppendorf, Hamburg, Germany). Glyceraldehyde 3-phosphate dehydrogenase (GAPDH) was amplified as the endogenous control. The primer sequences used were: GPC5: forward, 5′-CCCTCGAGGGAGGATGGACGCACAGACC-3′ and reverse, 5′-CGGGATCCCGCCAGGCATATGCAGA GAGAGAG-3′; GAPDH: forward, 5′-CAATGACCCC TTCATTGACC-3′ and reverse, 5′-TGGAAGATGGTGAT GGGATT-3′.

### Western blot analysis

Protein lysates were separated by 12% sodium dodecyl sulphate-polyacrylamide gel electrophoresis (SDS-PAGE), and electrophoretically transferred to a polyvinylidene difluoride (PVDF) membrane (Millipore, Billerica, MA, USA). Subsequently, the membrane was incubated with rabbit monoclonal antibody against human GPC5 (1:100; Abcam, Cambridge, UK) followed by horseradish peroxidase-labeled goat anti-rabbit IgG (Santa Cruz Biotechnology, Inc., Santa Cruz, CA, USA) and detected by chemiluminescence. GAPDH was used as a protein loading control. The intensity of protein fragments was quantified with ImageJ software (http://rsbweb.nih.gov/ij/).

### Wound scratch and transwell assays

Cells were seeded in 12-well plates and cultured overnight to form a confluent monolayer. After being scratched with a sterile pipette tip, the cells were rinsed gently with PBS to remove the detached cells and subsequently incubated with medium containing 1% FBS at 37°C in an atmosphere of 5% CO_2_. Images of the wounded areas were captured at 0 and 24 h after incubation. The distances between the two edges of the scratched cells were measured and the healing rate was calculated using the formula: healing rate = (the distance prior to healing - the distance following healing)/the distance prior to healing × 100. A Transwell (8 μm pore size; Costar, Corning, USA) assay was used to further analyze cell migration according to the manufacturer’s instructions. Fifty thousand cells were placed in the upper chambers in serum-free media, and the lower chambers were filled with RPMI-1640 + 10% FBS. Following incubation for 24 h at 37°C, non-migrating cells on the top surface of the membrane were removed with a cotton swab. The membranes were fixed with methanol for 10 min and stained with 0.5% crystal violet for 5 min. The number of cells that migrated to the bottom of the filter were counted manually under an inverted microscope.

### Study population and data collection

A panel of formalin-fixed paraffin-embedded (FFPE) NSCLC tissues from patients undergoing curative resection between January, 2003 and December, 2008 were obtained from the The Affiliated Drum Tower Hospital of Nanjing University Medical School (Nanjing, China). Full medical record abstraction was performed in order to obtain the following patient variables: age, gender, smoking status, respiratory symptoms upon lung cancer diagnosis, cell type, tumor differentiation, vascular invasion, regional lymph node metastasis, pTNM stage, adjuvant treatment and other medical conditions. Smokers were defined as those having smoked at least 100 cigarettes in their lifetime. pTNM staging designations were made according to the postsurgical pathological staging system according to the 7th edition of the TNM classification of malignant tumors (10). Complete removal of the primary lesion with negative resection margins was requested. All patients had an Eastern Cooperative Oncology Group (ECOG) performance status of 0 or 1. A total of 127 patients with complete data were identified in the current analysis. All patients enrolled in the study were newly diagnosed with NSCLC and none had received neoadjuvant chemotherapy, radiation therapy or immunotherapy prior to surgical therapy. Informed consent was obtained from each patient in this study. Their contact materials and the study protocol were reviewed and approved by the Ethics Committee of Drum Tower Hospital Institutional Review Board (Nanjing, China).

### Immunohistochemistry (IHC)

FFPE tissue samples were deparaffinized and rehydrated. The endogenous peroxydase activity was blocked with 3% hydrogen peroxide in methanol for 15 min. For antigen retrieval, the slides were boiled under pressure for 3 min in 10 mM citrate acid buffer (pH 6.0). Non-specific binding was blocked with 10% normal goat serum (Abcam) for 30 min at room temperature. The slides were subsequently incubated with a rabbit polyclonal antibody to GPC5 (1:100; Abcam) for a further 30 min at room temperature and incubated overnight at 4°C. The slides were then washed in Tris-buffered saline and incubated sequentially with anti-rabbit IgG (dilution 1:400; Dako, Carpinteria, CA, USA). Color was developed by 15 min incubation with 3,3′-diaminobenzidine. Samples were counterstained with hematoxylin, followed by dehydration and mounting. Negative controls were included by replacing the primary antibody with PBS. Normal human brain tissue was detected as a positive control. The quality of IHC was confirmed using H&E staining. The evaluation of immunostaining of these samples was performed by two trained pathologists (J Yang and FQ Meng) who were unaware of the clinical background of the samples. The intensity and percentage of positive cells were considered. Five visual fields were randomly observed, and 100 cells in each field were counted (magnification, ×400). Positive cells from the 100 tumor cells in each field were counted. Tumor cells with a brown cell membrane were considered to be positive and were scored as: 3+, strong; 2+, moderate; 1+, weak; and 0, no staining. The average percentages of positively stained tumor cells were classified as: 0 for 0%; 1 for 1–33%; 2 for 34–66%; and 3 for 67–100%. The intensity and percentage scores were multiplied to yield a composite score of 1–9 for each sample. Composite scores of 1–3 were defined as indicating a low GPC5 expression, while scores of 4–9 were considered to indicate a high GPC5 expression.

### Statistical analysis

Data from *in vitro* studies were expressed as the means ± SD and statistical significance was assessed by the Student’s t-test. Survival was assessed up to December 31, 2011. Overall survival (OS) was calculated as the period from the date of surgery for NSCLC until death. Patients who were alive at the last contact were censored. Associations between clinicopathological variables and GPC5 protein expression were examined using Pearson’s χ^2^ test for categorical variables (or Fisher’s exact test if any sample number was <5), and the Student’s t-test for continuous variables. Univariate Cox proportional hazard models were used to evaluate the prognostic impact on survival of all factors of interest. Factors were included in multivariate models if P<0.05 in the univariate analysis. Kaplan-Meier analysis was performed for survival curves and statistical significance was assessed using the log-rank test. All analyses were performed with SPSS software, version 16.0 (SPSS Inc., Chicago, IL, USA). All tests were two-sided and performed at a significance level of 0.05.

## Results

### Expression of GPC5 in NSCLC cell lines

**Figure 1:**
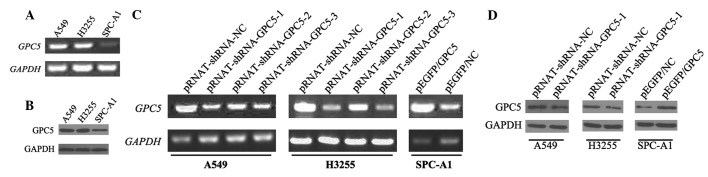
Glypican-5 (GPC5) expression in three non-small cell lung cancer (NSCLC) cell lines. (A and B) GPC5 expression in A549 and H3255 was higher than that in SPC-A1; (C and D) Expression of GPC5 was elevated in SPC-A1 following transfection of pEGFP/GPC5; GPC5 expression was downregulated following transfection with all three shRNAs. GAPDH, glyceraldehyde 3-phosphate dehydrogenase.

GPC5 expression in three NSCLC cell lines (two invasive: A549 and H3255, one less invasive: SPC-A1) were analyzed. GPC5 was highly expressed in A549 and H3255 cells, compared with SPC-A1 cells (Fig. 1A and B). Following transfection with pEGFP/GPC5, the expression of GPC5 was elevated in SPC-A1 (Fig. 1C and D). After screening three shRNA vectors, pRNAT-shRNA-GPC5-1 was identified as the most appropriate interfering plasmid for subsequent use. As indicated in Fig. 1C and D, pRNAT-shRNA-GPC5-1 may significantly downregulate the expression of GPC5.

### GPC5 enhances the migration ability of NSCLC cells

**Figure 2:**
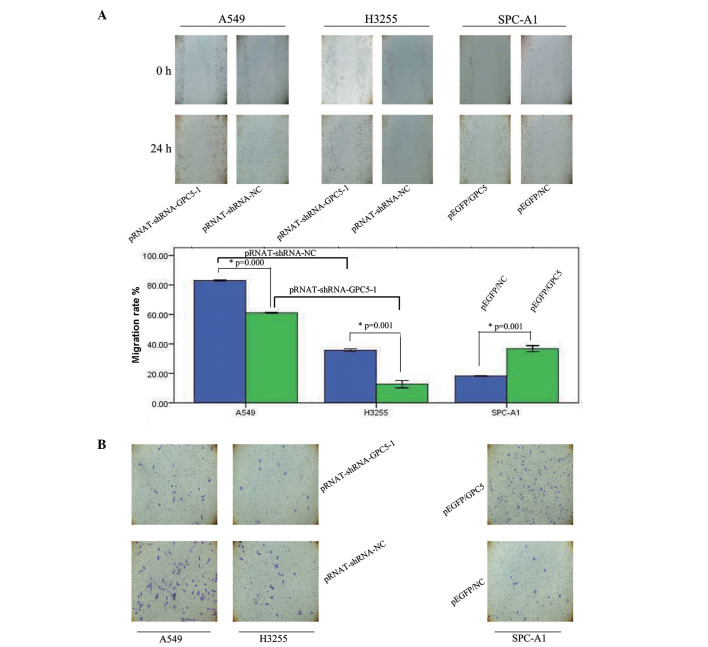
High glypican-5 (GPC5) expression levels lead to the increased motility of non-small cell lung cancer cells. (A) *In vitro* scratch assay, A549 and H3255 migrated markedly slower following transfection with pRNAT-shRNA-GPC5-1 compared with parental cells transfected with control vectors. SPC-A1 demonstrated a higher migration rate following transfection with pEGFP/GPC5. The histogram reveals significant changes to the cell migration rate within the time frame of the scratch experiment. (B) Transmigration was impeded in A549 and H3255 cells with the downregulation of GPC5 through pRNAT-shRNA-GPC5-1. Transmigration was promoted in SPC-A1 with the upregulation of GPC5 expression through pEGFP/GPC5.

Following transfection with pRNAT-shRNA-GPC5-1, A549 and H3255 cells migrated at a markedly slower rate than parental cells transfected with control vectors (Fig. 2A). The mean migration rate of A549 cells transfected with pRNAT-shRNA-GPC5-1 was (61±0.6%) while the mean rate of the control was (83±0.6%) (P<0.001). The mean migration rate of H3255 cells transfected with pRNAT-shRNA-GPC5-1 was (13±4.0%) while the mean rate of the control was (36±2.1%) (P=0.001). SPC-A1 with GPC5 overexpression demonstrated a higher migration rate than that of the control [(37±3.5%) vs. (18±0.6%), P=0.001] (Fig. 2A). Accordingly, the downregulation of GPC5 impeded the transmigration of A549 and H3255 cells from the top of the transwell membrane (Fig. 2B). The upregulation of GPC5 expression promoted transmembrane invasion compared with the control in SPC-A1 (Fig. 2B).

### Correlation between GPC5 protein expression and clinicopathological parameters in NSCLC

**Figure 3:**
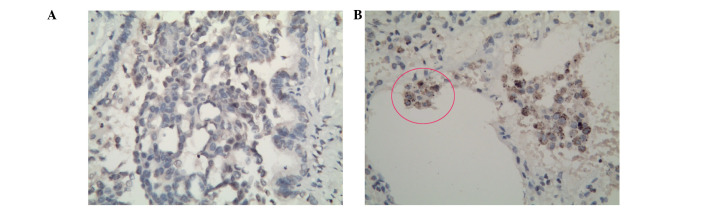
Microscopic views of positively stained immunohistochemical sections in non-small cell lung cancer. (Original magnification, ×400.) (A and B) Positive staining was mostly localized in the membranes of the tumor cells. (B) The red circle shows more-invasive tumor cells with high expression levels of GPC5.

Typical immunohistochemical staining patterns observed for GPC5 protein are shown in Fig. 3. Positive staining for GPC5 was mainly localized in the cell membrane. Tumor cells with high levels of GPC5 expression were more invasive compared to those with low levels of GPC5 expression (Fig. 3B). The median survival time of the 127 resected NSCLC patients was 33.0 months (95% CI: 20.65–45.35). The overall 5-year survival rate was 37.4%. High levels of GPC5 expression were detected in 58/127 (45.7%) of the NSCLC tissues. Clinicopathological characteristics of NSCLC are listed in Table I according to GPC5 expression status. There were no significant differences in age, gender, smoking status, cell type and adjuvant treatment between patients with high and low levels of GPC5 expression (P>0.05). Patients with high levels of GPC5 expression tended to have respiratory symptoms upon lung cancer diagnosis compared to those with low levels of GPC5 expression (P=0.03). Tumor cells with high GPC5 expression levels exhibited poor differentiation (P=0.04). The correlation between vascular invasion and GPC5 expression was also examined, with more cases demonstrating vascular invasion in the high GPC5 expression group (34.5 vs. 17.4%, P=0.03). A high GPC5 expression was found to correlate significantly with regional lymph node metastasis (P=0.007).

### Survival analysis

**Figure 4:**
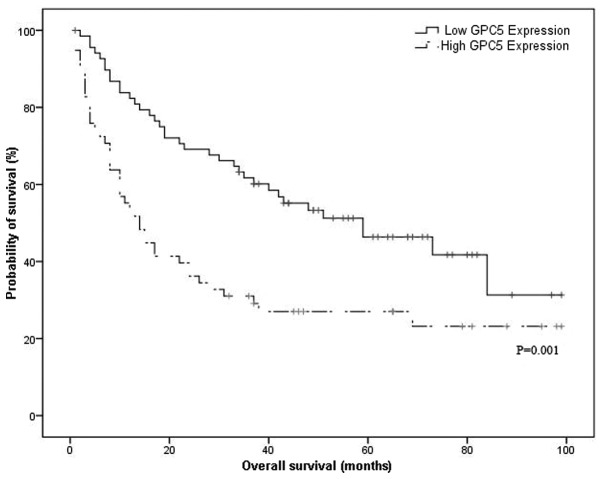
Overall survival in relation to glypican-5 (GPC5) expression levels. Patients with high levels of GPC5 expression had a shorter overall survival times [14.0 months (95% CI: 9.0–19.0) vs. 59.0 months (95% CI: 30.2–87.8), P=0.001].

The median OS time was 14.0 months (95% CI: 9.0–19.0) in patients with high levels of GPC5 expression and 59.0 months (95% CI: 30.2–87.8) in those with low levels of GPC5 expression, which demonstrated a significant difference (P=0.001, Fig. 4). For the univariate analysis, as shown in Table II, gender, respiratory symptoms, tumor differentiation, cell type, regional lymph node metastasis and stage all correlated significantly with OS (P<0.05). There was a significant correlation between GPC5 expression and OS (hazard ratio: 2.11; 95% CI: 1.35–3.30; P=0.001). Following adjustment for variables significant to univariate analysis, a significant correlation between GPC5 expression and OS remained, with a markedly larger effect (adjusted hazard ratio: 2.18; 95% CI: 1.35–3.52; P=0.001, data not shown).

## Discussion

In the present study, we identified GPC5 expression in NSCLC tumor cells and these expression levels may have affected tumor cell migration. To the best of our knowledge, this is the first study to investigate the role of GPC5 on NSCLC survival. High expression levels of GPC5 were identified as negative prognostic markers in patients with resected NSCLC.

The 8-exon GPC5 gene is located at chromosome 13q31.3, encoding a 572-amino acid product. The GPC5 protein belongs to the glypican gene family (GPC1-GPC6), the members of which have been reported to be overexpressed in several human malignancies (11). The function of GPC5 has yet to be established and studies of its role in tumors have been limited, although the 13q31–32 amplification has been observed in lung carcinomas (12), breast tumors (7), neurological tumors (8), liposarcomas (13) and rhabdomyosarcomas (9). In this study, GPC5-overexpressing NSCLC cells exhibited a high rate of migration, whereas GPC5 downregulated cells exhibited a low rate of migration. The enhancement of the migratory ability of cancer cells is an important factor in the promotion of tumor metastasis. Using IHC methods, high GPC5 expression levels were observed in patients with vascular invasion and regional lymph node metastasis. These findings suggest that GPC5 overexpression is likely a mechanism activated by NSCLC in order to promote cancer cell metastasis via vessels and lymph nodes, which requires confirmation with further molecular experiments. We explored the value of GPC5 as a molecular prognostic indicator and found that high levels of GPC5 expression predicted poor postsurgical survival times for curatively resected NSCLC patients.

These findings reveal the crucial role of GPC5 in NSCLC metastasis and survival. However, the mechanism responsible for GPC5-mediated effects on tumor metastasis remain to be clarified. Studies have highlighted potentially significant roles for the Wnt signaling pathway in the development of lung cancer in addition to being involved in mammalian limb development (14,15). The Wnt signaling pathway is also involved in numerous biological processes, such as cell proliferation, differentiation, survival, apoptosis and migration. In NSCLC, abnormal Wnt signaling has been observed (16,17). Certain Wnt proteins are expressed abnormally in NSCLC samples, including Wnt1, −2 and −7a. Wnt1 and Wnt2 were overexpressed in NSCLC samples and cancer cells, with Wnt1 expression exhibiting resistance to apoptosis-inducing therapy (18). Conversely, inhibiting Wnt1 and Wnt2 may lead to the apoptosis of cancer cells and decrease tumor growth *in vivo* and *in vitro*(17). As the Wnt pathway is involved in epithelial-mesenchymal transition (EMT), the activation of the Wnt signaling pathway may enhance the invasiveness of tumor cells (19). A study by Williamson *et al*(9) revealed that GPC5 overexpression increased proliferation in rhabdomyosarcoma by potentiating the effects of Wnt1. We hypothesize that GPC5 may promote tumor cell EMT by facilitating the activation of the Wnt pathway, which subsequently may enhance tumor invasiveness and metastasis, which requires further studies.

A recent GWAS suggested that genetic variants at 13q31.3 modulate GPC5 expression that had been downregulated in the adenocarcinomas in patients who had never smoked. The GPC5 expression levels were reduced in the mammary tumors of breast cancer patients (20). Associations of GPC5 in distinct phenotypes suggests that GPC5 may have multiple roles in different diseases. There may be numerous genes and modulators involved in GPC5 gene expression, which should be incorporated into the future studies.

In conclusion, our findings suggest that the evaluation of GPC5 expression may be useful clinically in recognizing patients who are more likely to have a poor NSCLC outcome. Specifically, higher levels of GPC5 expression suggest a tendency for a shorter survival time. Furthermore, GPC5 may be involved in NSCLC metastasis through enhancing cancer cell migration.

## Figures and Tables

**Table I:** Characteristics of 127 non-small cell lung cancer patients.

	GPC5 expression		
		
Patient characteristics	High [No. (%)]	Low [No. (%)]	P-value
Total patients	58	69	
Age (years)			0.93
Mean (SD)	63.45 (11.61)	63.28 (11.32)	
Gender			0.97
Female	17 (29.3)	20 (29.0)	
Male	41 (70.7)	49 (71.0)	
Respiratory symptoms			0.03
No	12 (20.7)	27 (39.1)	
Yes	46 (79.3)	42 (60.9)	
Smoking status			0.31
Never smokers	25 (43.1)	36 (52.2)	
Smokers	33 (56.9)	33 (47.8)	
Tumor differentiation			0.04
Well + moderate	31 (53.4)	49 (71.0)	
Poorly	27 (46.6)	20 (29.0)	
Cell type			0.92
Adenocarcinoma	30 (51.7)	36 (27)	
Squamous	24 (41.4)	27 (39.1)	
Others^a^	4 (6.9)	6 (8.7)	
Vascular invasion			0.03
No	38 (65.5)	57 (82.6)	
Yes	20 (34.5)	12 (17.4)	
Regional lymph node metastasis			0.007
No	23 (39.7)	44 (63.8)	
Yes	35 (60.3)	25 (36.2)	
Stage			0.003
I	18 (31.0)	37 (53.6)	
II	12 (20.7)	18 (26.1)	
III	28 (48.3)	14 (20.3)	
Adjuvant chemotherapy and/or radiation			0.32
No	37 (63.8)	38 (55.1)	
Yes	21 (36.2)	31 (44.9)	

GPC5, glypican-5; SD, standard deviation.

a Includes large cell and adenosquamous carcinoma.

**Table II:** Univariate analysis.

Patient characteristics	Unadjusted HR (95% CI)	P-value
Age (years)		
<65	Reference	
>65	1.12 (0.72–1.74)	0.62
Gender		
Female	Reference	
Male	1.74 (1.02–2.98)	0.04
Respiratory symptoms		
No	Reference	
Yes	1.80 (1.06–3.05)	0.03
Smoking status		
Never smokers	Reference	
Smokers	1.39 (0.89–2.17)	0.15
Tumor differentiation		
Well + moderate	Reference	
Poorly	1.91 (1.23–2.99)	0.004
Cell type		
Adenocarcinoma	Reference	
Others^a^	1.85 (1.18–2.89)	0.007
Vascular invasion		
No	Reference	
Yes	1.51 (0.92–2.45)	0.10
Regional lymph node metastasis		
No	Reference	
Yes	2.17 (1.38–3.41)	0.001
Stage		
I	Reference	
II	2.53 (1.42–4.52)	0.002
III	2.71 (1.57–4.66)	<0.001
Adjuvant chemotherapy and/or radiation		
No	Reference	
Yes	1.00 (0.64–1.56)	1.00
GPC5 expression level		
Low	Reference	
High	2.11 (1.35–3.30)	0.001

HR, hazard ratio; CI, confidence interval; GPC5, glypican-5.

a Includes squamous cell carcinoma, large cell and adenosquamous carcinoma.
